# The effectiveness of online therapy in promoting wellbeing and reducing burnout among psychotherapists

**DOI:** 10.3389/fpsyg.2025.1510383

**Published:** 2025-03-31

**Authors:** Maria Valentina Cavarretta, Hugues Pellerin, Ema Maurel, Salvatore Maria Anzalone, Isis Truck, David Cohen, Sonia Ingoglia

**Affiliations:** ^1^Department of Psychology, Educational Science and Human Movement, University of Palermo, Palermo, Italy; ^2^Laboratoire de Cognitions Humaine et Artificielle, CHArt, Université Paris 8, Saint-Denis, France; ^3^Département de Psychiatrie de l’Enfant et de l’Adolescent, AP-HP, Hôpital Pitié-Salpêtrière, Paris, France; ^4^Laboratoire de Cognitions Humaine et Artificielle, CHArt, CY Cergy Paris Université, Paris, France; ^5^Institut des Systèmes Intelligents et Robotiques, CNRS UMR 7222, Sorbonne Université, Paris, France

**Keywords:** burn-out, online therapy, telepsychology, environmental sensitivity, clinical method, quality of care, mental health strategy

## Abstract

**Introduction:**

The COVID-19 pandemic has worsened global mental health, thereby burdening mental health services and raising burnout risk among professionals. Online therapy may be an optimal solution to reduce burnout risk, ensuring flexibility for psychotherapists and the continuity of care for patients. This study investigates the link between burnout and online therapy, focusing on environmental sensitivity and exploring tailored solutions to reduce burnout while maintaining healthcare performance.

**Method:**

Participants were 95 French psychotherapists (89% females), aged from 24 to 59 years (*M* = 37.13, SD = 7.75). Participants were administered the Maslach Burnout Inventory, the Highly Sensitive Person Scale, and a questionnaire assessing their professional activity.

**Results:**

Digital psychotherapists reported lower levels of burnout compared to traditional psychotherapists who did not use online therapy. Specifically, they had lower depersonalization scores (mean difference of 0.37 points, *p* = 0.038) and tent to have lower scores in emotional exhaustion (mean difference of 0.44 points, *p* = 0.07). This association was more pronounced for those with high environmental sensitivity.

**Discussion:**

Online therapy ensures greater workplace flexibility, serving as a protective factor in reducing psychotherapists’ burnout. Integrating digital health into public mental health services can enhance care delivery and support the wellbeing of professionals, especially those with high environmental sensitivity. Essential guidelines for online therapy use are needed to maximize benefits and ensure its effective implementation.

## 1 Introduction

Interruptions in mental health services worldwide during the COVID-19 pandemic and the subsequent surge in the demand for psychological support have exacerbated the risk of burnout among mental health professionals, with negative outcomes for entire healthcare organizations (Clemente-Suárez et al., 2021; Cohen et al., 2024; Gruber et al., 2021; Joshi and Sharma, 2020; Kane et al., 2022; Kupcova et al., 2023).

Most recent literature focuses on the psychological distress of healthcare professionals who were at the frontline during the pandemic (Leo et al., 2021; Ulfa et al., 2022). As a result, the wellbeing of “second-line” operators has often been overlooked (Billings et al., 2021). Some studies have reported prolonged and chronic workplace stress and high levels of burnout among mental health workers compared to other healthcare professionals (Franzoi et al., 2021), and burnout is particularly common in helping professions (Maslach et al., 2001). However, unlike other healthcare occupations (e.g., doctors or nurses), research on burnout among psychotherapists is significantly less prevalent (Schaufeli et al., 2009; Woo et al., 2020; Van Hoy and Rzeszutek, 2022).

Among psychotherapists, occupational burnout, characterized by emotional exhaustion, depersonalization, and reduced personal accomplishment, negatively affects the clinical process and patient outcomes, potentially resulting in partial or complete therapy failure (Vivolo et al., 2024). Research indicates that unmanaged burnout among clinicians can contribute to reduced empathy and diminished clinical effectiveness in psychotherapy and, in some cases, even lead to unethical conduct (Anagnostopoulos et al., 2012; Simionato et al., 2019). This is unfortunate as clinical work with patients and the continuous interaction with individuals experiencing physical and social suffering require a high degree of emotional and relational investment (Holmqvist and Jeanneau, 2006). This investment can lead to emotional exhaustion in therapists, expressed as decreased energy, stress, fatigue, and irritability, potentially culminating in withdrawal. It can also manifest as depersonalization, expressed as detachment from others, cynicism, and disengagement. Burnout may hinder a professional’s ability to establish a therapeutic alliance with patients (Zarzycka et al., 2022). As the therapeutic alliance is the cornerstone of the clinical method, personal fulfillment related to clinical work may decline (Stubbe, 2018).

Several studies have highlighted the significant role of intrapersonal variables as predictors of individual burnout (Mills and Huebner, 1998; Rzeszutek and Schier, 2014; Lee et al., 2016; George-Levi et al., 2020; Smout et al., 2021).

Some individuals appear to be more at risk of burnout due to their sensitivity to environmental influences, while others show greater resilience to stress (Golonka and Gulla, 2021; Rossi et al., 2023). Individuals with heightened environmental sensitivity, due to deeper perception and processing of stimuli, are better at recognizing details of their environment and are more strongly influenced by what happens around them (Belsky, 2013). Consequently, they have the potential to be more deeply affected by stressful experiences (Pluess, 2015; Pluess et al., 2023).

The theoretical concept of environmental sensitivity has important implications for intervention strategies in various settings, including workplaces (Lionetti et al., 2024; Nocentini et al., 2018). People differ significantly in their response to interventions, and some benefit more than others; part of this variability may stem from individual differences in environmental sensitivity (also known as “vantage sensitivity”) (Pluess, 2017; Pluess and Belsky, 2013).

Although there are numerous studies on the personality-burnout association, this issue remains understudied in the specific occupation of psychotherapists (Van Hoy and Rzeszutek, 2022). Several studies have revealed significant associations between high workload in the public sector and increased burnout levels (Ibrahim et al., 2022; Rupert and Dorociak, 2019; Rupert et al., 2009). This evidence is often explained by the lack of control over the work environment among psychotherapists (Kim, 2017). For these reasons, it is necessary to implement personalized burnout prevention interventions aimed at Mental Health Professionals (Johnson et al., 2018; Kotera et al., 2021; Santillan-Ramos et al., 2024).

In this sense, teletherapy, in the form of video conferencing for clinical practice, may be an optimal solution to address burnout, promoting therapists’ wellbeing while simultaneously meeting patients’ bio-psycho-social treatment needs (Bhaskar et al., 2020; Patel et al., 2018; Saladino et al., 2020; Tajan et al., 2023). Teletherapy has been defined as the provision of mental health care at a distance through technology (Shore, 2015; Wibberly, 2024).

Teletherapy aligns with the goals of U.S. healthcare reform to increase “effective, cost-efficient, and patient-centered practices” (Backhaus et al., 2012).

Remote therapy can support psychotherapists by improving their working conditions (Feijt et al., 2020; Kane et al., 2022). Online consultations may help alleviate clinicians’ stress by providing them with more free time while maintaining the quality of care provided to clients (Al-Mahrouqi et al., 2022; Harris et al., 2023).

Reduced commuting time for psychotherapists increases efficiency and enables the immediate provision of care across different clinical settings and geographic regions (Chevillard et al., 2018).

Telepsychiatry improves flexibility for professionals who can deliver care from home, even when living or traveling out of state or abroad (Gardner et al., 2020). Additional advantages of telepsychiatry include reduced travel costs, increased educational opportunities, earlier interventions, bundled services, better care coordination, and access to patients in non-medical settings such as schools, childcare facilities, prisons, and homes (Simpson and Reid, 2014).

Furthermore, the support of digital resources ensures consistent, equitable, and uninterrupted access to mental health services, particularly in underserved rural areas (Poletti et al., 2021; Sampaio et al., 2021; Tohme et al., 2021). This innovative strategy optimizes clinical workflows with timely patient engagement (Mitchell et al., 2021; Stefan et al., 2021).

Recent reviews of empirical data indicate that patients and clinicians using online therapy via video conferencing generally develop a good therapeutic alliance (Békés and Aafjes-van Doorn, 2020; Simpson and Reid, 2014), and online sessions do not differ in effectiveness from in-person sessions (Backhaus et al., 2012; Fernandez et al., 2021; Shklarski et al., 2021; Simpson, 2009).

In conclusion, the pandemic has accelerated innovations, including the adoption of new laws and regulations to promote the integration of online therapy into clinical practice (Bhaskar et al., 2020). Given the benefits and positive data on online therapy, many companies and healthcare organizations, as well as individual professionals, use this approach to support the profound and complex work of psychotherapy and as a sustainable practice to ensure continuity of care (Conrad, 2024; Jurcik et al., 2020; Peng et al., 2020).

### 1.1 Aims of the study

This exploratory study aims to explore burnout among psychotherapists whether they use or not online therapy, to evaluate potential risk and protective factors associated with burnout including environmental sensitivity. To do so, we first examined the association of burnout with age, gender, and some characteristics of their professional activity (years of clinical experience, sector of professional practice, and therapeutic approach). Second, we explored the association of burnout with using online therapy. Third, we examined the association between burnout and environmental sensitivity. Finally, we tested the possible moderating effect of environmental sensitivity in the relationship between the use of online therapy and burnout.

## 2 Materials and methods

### 2.1 Participants

The study was carried out on a sample of 95 psychotherapists, (89% females), ranged in age from 24 to 59 years (*M* = 37.13, SD = 7.75). The inclusion criteria for participants were as follows: (a) Working in mental health care during and after the COVID-19 outbreak; (b) providing informed consent.

The sample included psychotherapists who conducted online therapy (57%) and others who did not practice online therapy (43%). Among digital psychotherapists, 51% had been conducting online therapy for 1–4 years, and 4% for 5–8 years. Most participants were psychologists (84%), with an equal distribution of child psychiatrists, neuropsychologists, and psychomotor therapists among the other participants.

The majority of participants practiced their profession in metropolitan France (70.1%). The rest were a minority, distributed as 1% in Belgium, 2% in overseas France. Information was not available for 27% of the participants, that have chosen not to respond.

One third (33%) of psychotherapists had an integrative approach, which included transactional analysis, cognitive-behavioral therapy, psychoanalysis, and body therapies, 22% had a psychodynamic approach, which included psychoanalysis, group analysis and family therapy, 16% employed a cognitive-behavioral approach alone, 29% did not specified their approach.

The majority of participants worked in the private sector (59%), had an average of 8.8 years of clinical experience (SD = 6.60) (Table 1).

**Table 1:** Participants’ description.

Socio-demographic characteristics	*N* = 95
Age^a^	37.13 (7.75) [24,59]
N/A	2
Gender^b^	
Female	85 (89%)
Male	10 (11%)
Other gender	0
Country of professional practice^b^	
Metropolitan France	68 (70.1%)
Overseas France	2 (2.06%)
Belgium	1 (1.03%)
N/A	26 (26.8%)
Sector of professional practice^b^	
Private	56 (59%)
Public	39 (41%)
Clinical experience^c^	8.80 (6.60)
N/A	8
Use of online therapy^b^	
Yes	54 (57%)
No	40 (43%)
Experience of online therapy^b^	
1–4 years	49 (50.5%)
5–8 years	4 (4.12%)
N/A	44 (45.4%)
Psychotherapeutic approach^b^	
Psychodynamic	21 (21.65%)
Integrative Therapy	32 (32.99%)
Cognitive-Behavioral Therapy	16 (16.49%)
Other	2 (2.06%)
N/A	26 (26.8%)

^a^Mean (SD) [range]; ^b^N (%); ^C^mean (SD).

### 2.2 Procedure

The study was conducted from February to June 2024. A questionnaire, developed using the Google® Forms platform, was disseminated to potential participants via a link. This link to the survey was sent directly to psychotherapists working in mental health services, psychiatric hospitals and psychological medical centers in France. It was also sent to professionals registered on Doctolib.fr, a French digital health platform facilitating online psychological appointment booking and teleconsultations between patients and healthcare providers. Additionally, an announcement containing instructions and the survey link was also disseminated through social media professional networks. Prior to participation, all respondents were presented with an online informed consent form. The survey was anonymous, and participants were assured of the confidentiality of their responses. Informed consent was obtained electronically from all participants before they could access the survey questions.

The study protocol and informed consent procedures were approved by the Ethics Committee of the University of Palermo (Ref 3145/2022, November 11th, 2022) prior to the commencement of data collection. The study was conducted in adherence to the Helsinki Declaration.

### 2.3 Measures

#### 2.3.1 Demographic characteristics and professional activity

We collected participants’ age and gender, information on professional experience, including the country and sector of professional practice (public or private), the years of clinical experience, the clinical practice modality (use or non-use of online therapy), years of experience with providing online therapy and the psychotherapeutic approach.

#### 2.3.2 Burnout

To measure participants’ levels of professional burnout, we used The French version of the Maslach Burnout Inventory - Human Service Survey (MBI-HSS; Lheureux et al., 2017). It is a 22-item questionnaire designed to assess burnout in individuals working in healthcare and social services. The MBI-HSS comprises three subscales: Emotional Exhaustion (EE, 9 items, e.g., “*Working with people all day is really a strain for me*”) which measures feelings of being emotionally overextended by one’s work; Depersonalization (DP, 5 items, e.g., “*I do not really care what happens to some patients*”) which assesses unfeeling and impersonal responses to care and Personal Accomplishment (PA, 8 items, e.g., “*I have accomplished many worthwhile things in this job*”), which evaluates feelings of competence and perceived effectiveness in the job.

Respondents indicate the frequency with which they experience specific feelings or attitudes using a 7-point Likert scale, ranging from 0 (*never*) to 6 (*every day*).

The scores for each subscale are reported separately and are not combined into a global score.

In the current study, the scale showed an acceptable internal consistency, with Cronbach’s *α* values of 0.88 for EE, 0.60 for DP, and 0.79 for PA.

#### 2.3.3 Environmental sensitivity

To measure the degree of an individual’s sensitivity to environmental influences, we used the Highly Sensitive Person scale (HSP-12; Pluess et al., 2023). It is a 12-item scale and includes three subscales: Ease of Excitation (EOE, 5 items, e.g., “*Do you get rattled when you have a lot to do in a short amount of time?*”) which refers to being easily overwhelmed by external and internal demands; Aesthetic Sensitivity (AES, 4 items, e.g., “*Do you seem to be aware of subtleties in your environment?*”) which captures the response and appreciation of aesthetic stimuli and Low Sensory Threshold (LST, 3 items, e.g., “*Are you bothered by intense stimuli, like loud noises or chaotic scenes?*”) which reflects unpleasant arousal to external stimuli. Respondents were asked to answer using a 7-point Likert scale, ranging from 1 (*Not at all*) to 7 (*Completely*).

In the current study, the scale showed a good internal consistency, with Cronbach’s α values of 0.82 for EOE, 0.72 for AES, and 0.72 for LST.

### 2.4 Statistical analysis

Analyses were performed using R software version 4.2.2. A *p*-value <0.05 was considered significant; however, we encourage the reader to interpret them here as a continuous measure of evidence against the null hypothesis.

The distribution of quantitative variables was summarized using mean and standard deviation, and alternatively using median and Q1–Q3 quartiles depending on the shape of the distribution. The distribution of qualitative variables was described using absolute and relative frequencies.

To explore psychotherapists’ workplace stress, we looked for factors associated with the three-dimensional scores of the degree of burnout. The association between the scores and quantitative variables was tested using Pearson correlation tests. The association between the scores and binary variables was tested using Welch *t-*tests or, alternatively, Wilcoxon rank sum tests, depending on the validity of the tests’ assumptions. For nominal variables, a Kruskal–Wallis test was used.

Finally, we tested the hypothesis that environmental sensitivity (as measured by three dimensional scores of the HSP scale) could moderate the association between using online therapy and the degree of burnout. This was done using linear regression models of the formula “Burnout ~ use of online therapy (yes/no) + environmental sensitivity + use of online therapy * environmental sensitivity.” One model was run for each combination of burnout score and environmental sensitivity subscore. We report for each model the effect of the interaction, tested using a *t*-test, along with its 95% parametric confidence interval.

## 3 Results

### 3.1 Descriptive statistics

Regarding burnout, testimated average level of Emotional Exhaustion (EE) was 2.07 Likert points, corresponding to “once a month” (SD =1.13; 95% CI 1.85; 2.30). There was a variability, with scores ranging from 0 (“never”) to 5.11 Likert points (“a few times a week”). Half of the participants scored between 1.22 and 2.78 Likert points.

The average Depersonalization (DP) level is 0.96 Likert points, indicating occurrences of “a few times a year” (SD = 0.85; 95% CI 0.78; 1.13). The DP score ranged from 0 to a maximum of 3.60 Likert points.

Conversely, Personal Accomplishment (PA) showed higher levels, with an average of 4.62 Likert points, suggesting a frequency between “once a week” and “a few times a week” (SD = 0.80; 95% CI 4.46; 4.78; Kurt = 2.68; Skew = −1.34). Scores in this domain ranged from 1.75 to 5.88 Likert points.

Regarding environmental sensitivity, the estimated average level of Ease of Excitation (EOE) was 4.18 Likert points (SD = 1.32), with scores ranging from 0 1.20 to 7.00.

The mean Aesthetic Sensitivity (AES) level was 5.56 Likert points (SD = 0.97), with scores ranging from 2.75 to 7.00 Likert points.

Finally, the estimated average level of Low Sensory Threshold (LST) was 3.86 Likert points (SD = 1.59), with scores ranging from 1.00 to 7.00 Likert points.

### 3.2 Associations of burnout with demographic characteristics and professional activity

Results are reported in Table 2. Significant associations between burnout and gender were found. Female psychotherapists reported significantly higher EE scores, with a difference of 0.83 points between the median scores (*p* = 0.026). Additionally, they showed higher PA scores, with a difference of 0.5 points between the median scores, approaching significance (*p* = 0.078).

**Table 2:** Correlation analysis between burnout scores, demographic and professional characteristics.

	Emotional exhaustion (EE)	Depersonalization (DP)	Personal accomplishment (PA)
Age ^a^			
	*r* = −0.04	*r* = 0.03	*r* = 0.03
	*p* = 0.710	*p* = 0.739	*p* = 0.795
	*N* = 95	*N* = 95	*N* = 95
Gender ^b^			
Female	2.11[1.33;2.78]	0.8 [0.2;1.4]	4.88 [4.25;5.12]
Male	1.28 [0.75;1.67]	0.9 [0.45;1.95]	4.38 [4.12;4.72]
	*N* = 95, *p* = 0.026 *	*N* = 95, *p* = 0.393	*N* = 95, *p* = 0.078
Sector of professional practice ^c^			
Private	1.91 (1.06)	0.84 (0.78)	4.7 (0.75)
Public	2.3 (1.2)	1.13 (0.93)	4.52 (0.86)
	*N* = 95, *p* = 0.104	*N* = 95, *p* = 0.112	*N* = 95, *p* = 0.303
Clinical experience ^a^			
	r = 0.07	r = 0.06	*r* = −0.06
	*p* = 0.534	*p* = 0.591	*p* = 0.582
	*N* = 89	*N* = 89	*N* = 89
Psychotherapeutic approach ^b^			
Psychodynamic	2.44 [1.89;3.44]	0.8 [0.2;1.8]	4.62 [4.12;5]
Integrative therapy	1.94 [1.31;2.78]	0.9 [0.2;1.7]	4.88 [4.12;5.25]
Cognitive-behavioral	2.17 [1.08;3.25]	1 [0.4;1.8]	4.94 [4.34;5.25]
	*N* = 69, *p* = 0.225	*N* = 69, *p* = 0.926	*N* = 69, *p* = 0.298

^a^ Pearson correlation coefficient, *p*-value, participants number; ^b^ median[q1,q3]; ^c^ mean(SD).

With regard to age, sector of professional practice, years of clinical experience and psychotherapeutic approach, no significant associations were found.

### 3.3 Associations between burnout and the use of online therapy

Results are reported in Table 3. Significant associations between burnout and the use of online therapy were found. Participants who utilized online therapies showed lower scores in EE, with a mean difference of 0.44 points approaching statistical significance (*p* = 0.07) compared to participants who did not use online therapy. Moreover, psychotherapists who practiced online therapy exhibited significantly lower DP scores, with a mean difference of 0.37 points (*p* = 0.038) compared to psychotherapists who did not use online therapy.

**Table 3:** Correlation analysis between burnout scores and the use of online therapy.

	**Emotional Exhaustion (EE)**	**Depersonalization (DP)**	**Personal Accomplishment (PA)**
Use of online therapy ^a^			
Yes	1.89 (1.06)	0.8 (0.81)	4.7 (0.86)
No	2.33 (1.19)	1.17 (0.88)	4.52 (0.72)
	*N* = 94, *p* = 0.07	*N* = 94, *p* = 0.038 *	N = 94, *p* = 0.267
Frequency of online therapy use ^b^			
	r = −0.03	r = −0.05	*r* = −0.08
	*p* = 0.821	*p* = 0.698	*p* = 0.549
	*N* = 55	*N* = 55	*N* = 55

^a^ Mean(SD); ^b^ Pearson correlation coefficient; *p*-value, participants number.

### 3.4 Associations between burnout and environmental sensitivity

Results are reported in Table 4. Highly significant positive correlations were found between EE and both EOE (*r* = 0.35, *p* = 0.001) and LST (*r* = 0.30, *p* = 0.003). A small negative correlation was observed between PA and EOE, approaching significance (*r* = −0.20, *p* = 0.055). Finally, a positive correlation was observed between PA and AES (*r* = 0.31, *p* = 0.002).

**Table 4:** Correlation analysis between burnout scores and environmental sensitivity scores.

	Emotional exhaustion (EE)	Depersonalization (DP)	Personal accomplishment (PA)
Ease of excitation (EOE) ^a^	*r* = 0.35	*r* = 0.05	*r* = –0.20
	*p* = 0.001***	*p* = 0.618	*p* = 0.055
	*N* = 95	*N* = 95	*N* = 95
Aesthetic sensitivity (AES) ^a^	*r* = 0.07	*r* = –0.07	*r* = 0.31
	*p* = 0.518	*p* = 0.529	*p* = 0.002**
	*N* = 95	*N* = 95	*N* = 95
Low sensory threshold (LST) ^a^	*r* = 0.30	*r* = 0.03	*r* = 0.00
	*p* = 0.003**	*p* = 0.781	*p* = 0.999
	*N* = 95	*N* = 95	*N* = 95

^a Pearson correlation coefficient, p-value.^

DP was not significantly related with environmental sensitivity dimensions.

### 3.5 Environmental sensitivity as a moderator of the relation between burnout and online therapy use

**Figure 1:**
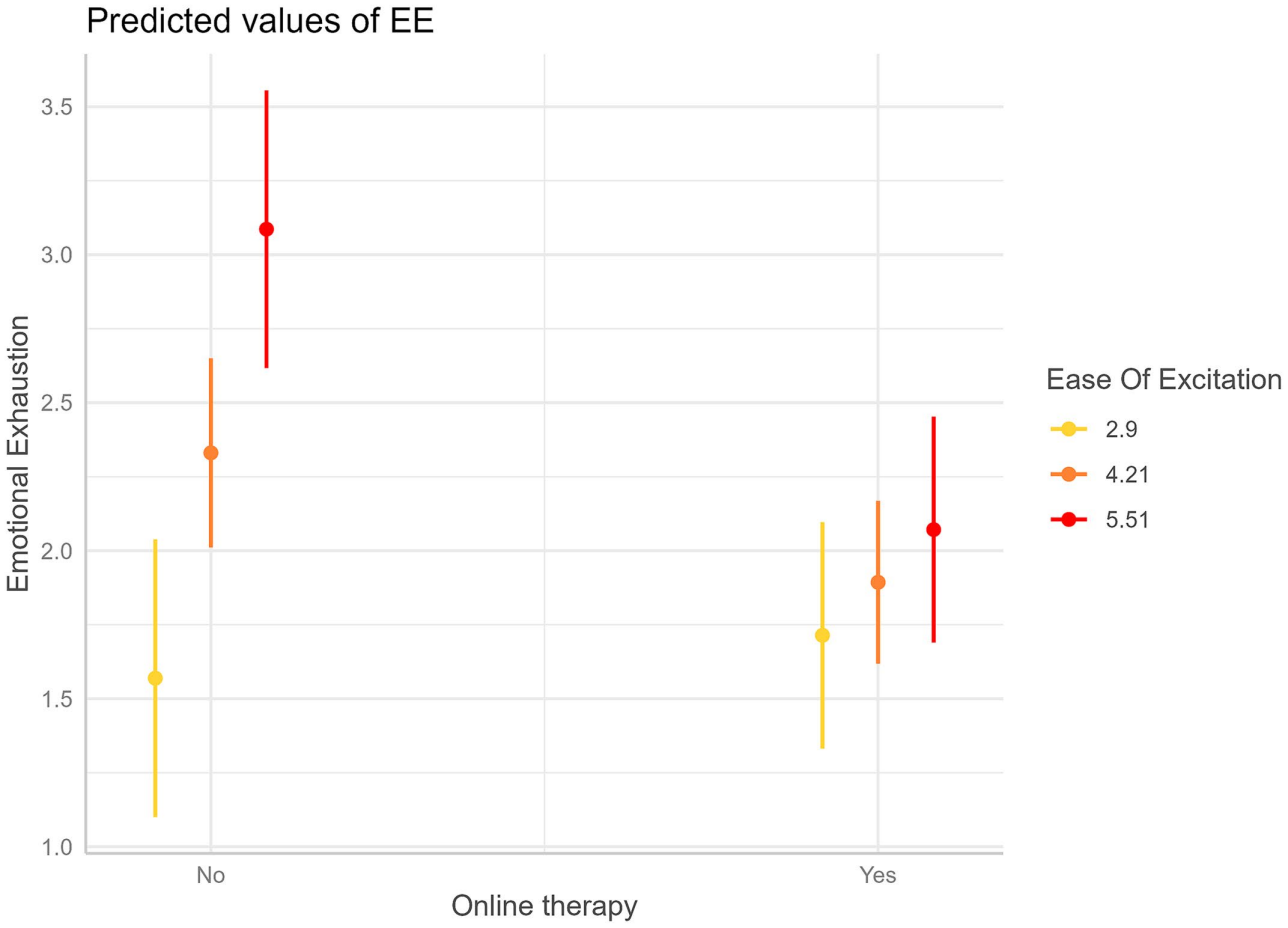
Interaction plot—Predicted values of Emotional Exhaustion (EE) as a function of online therapy use (Yes/No) at three values of Ease of Excitation (EOE), represented in colors. Error bars are 95% confidence intervals. This graph shows that a higher degree of EOE is associated with a higher reduction of EE for psychotherapists conducting online therapies compared to those who are not.

**Figure 2:**
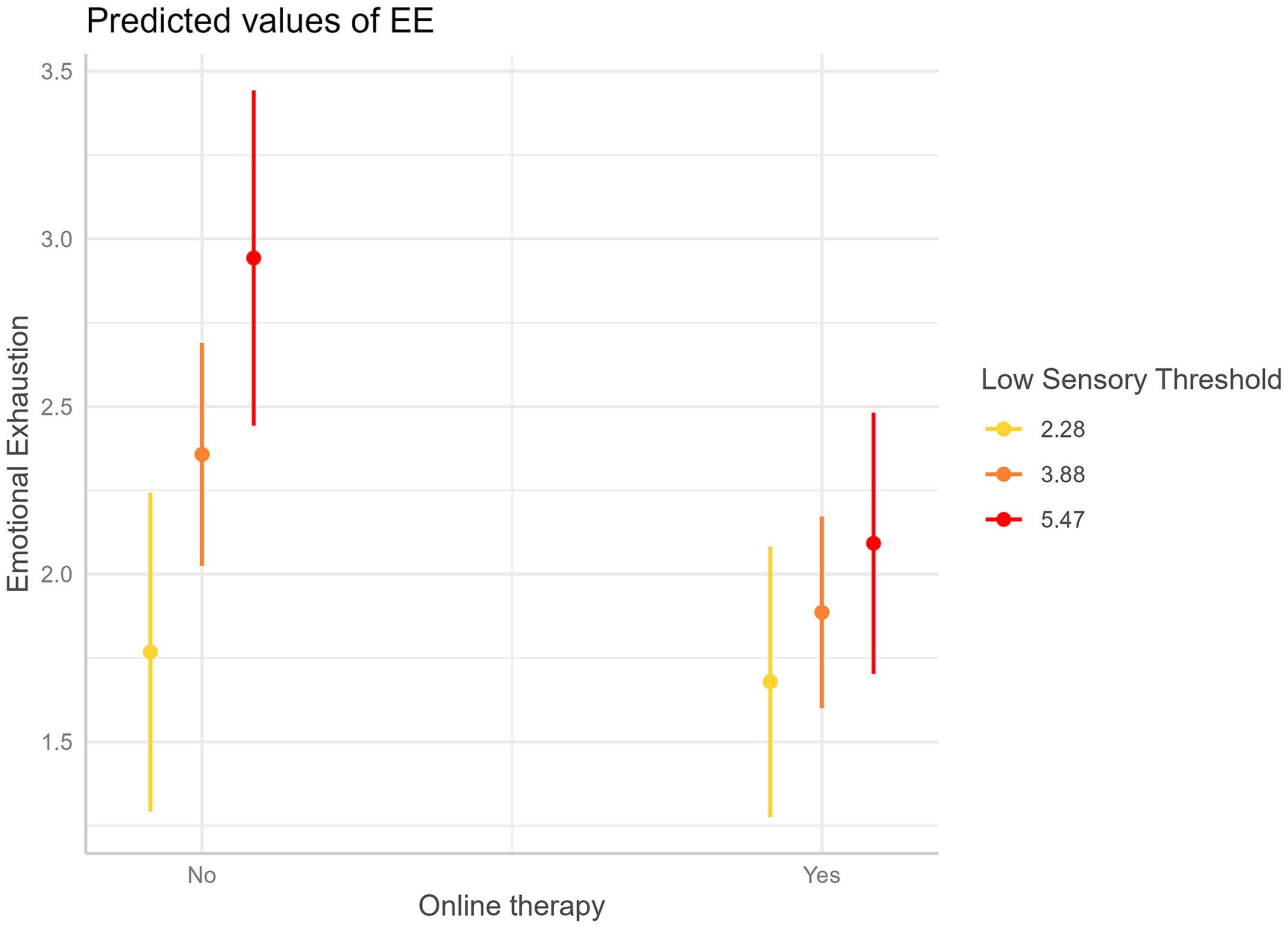
Interaction plot—Predicted values of Emotional Exhaustion (EE) as a function of online therapy use (Yes/No) at three values of Low Sensory Threshold (LST), represented in colors. Error bars are 95% confidence intervals. This graph shows that a higher degree of LST is associated with a higher reduction of EE for psychotherapists conducting online therapies compared to those who are not.

Results are reported in Table 5 and Figures 1, 2. The analysis revealed that EOE negatively moderates the association between online therapy use and EE (Beta = −0.44, *p* = 0.009).

**Table 5:** Analysis of environmental sensitivity scores dimensions as moderators of the association between online therapy use and burnout scores.

Outcome	Parameter	Coefficient	SE	95%CI_low	95%CI_high	*p*-value	*N*
Emotional exhaustion (EE)	Online therapy (yes): Ease of excitation (EOE)	−0.44	0.17	−0.78	−0.11	0.009**	94
Emotional exhaustion (EE)	Online therapy (yes): Aesthetic sensitivity (AES)	−0.26	0.24	−0.74	0.22	0.281	94
Emotional exhaustion (EE)	Online therapy (yes): Low sensory threshold (LST)	−0.24	0.14	−0.52	0.04	0.097	94
Depersonalization (DP)	Online therapy (yes): Ease of excitation (EOE)	−0.21	0.14	−0.48	0.07	0.134	94
Depersonalization (DP)	Online therapy (yes): Aesthetic sensitivity (AES)	−0.08	0.18	−0.44	0.29	0.682	94
Depersonalization (DP)	Online therapy (yes): Low sensory threshold (LST)	0.01	0.11	−0.22	0.24	0.934	94
Personal accomplishment (PA)	Online therapy (yes): Ease of excitation (EOE)	0.16	0.13	−0.10	0.42	0.227	94
Personal accomplishment (PA)	Online therapy (yes): Aesthetic sensitivity (AES)	0.24	0.17	−0.09	0.56	0.159	94
Personal accomplishment (PA)	Online therapy (yes): Low sensory threshold (LST)	0.17	0.11	−0.05	0.38	0.127	94

**Indicates that the result is significant.

For example, for an EOE of 2.90 points Likert, online therapy use does not show a significant association with EE. However, for an EOE of 4.21 points Likert, a significant negative association begins to appear with a decrease in EE of 0.44 Likert points (*p* = 0.042) for psychotherapists who use online therapy compared to psychotherapists who do not use it. The negative association becomes more pronounced at higher levels of EOE. Finally, for an EOE of 5.51 points, the negative association becomes highly significant with an average decrease in EE of 1.01 Likert points (*p* = 0.001) when the psychotherapists use teleconferences compared to when they do not use them.

The analyses revealed also that the association between online therapy use and EE tended to decrease with LST (Beta = −0.24, *p* = 0.097), although this relationship approached but did not reach statistical significance.

For example, at lower levels of LST (2.28 Likert points), online therapy use does not show a significant association with EE. However, as LST increases, a negative association begins to emerge. Specifically, at an LST of 3.88 Likert points, a significant reduction in EE of 0.47 Likert points is observed (*p* = 0.036) when psychotherapists conduct teleconsultations compared to those who do not. This negative association becomes more pronounced at higher levels of LST. For instance, at an LST of 5.47 Likert points, psychotherapists conducting online therapy experience a substantial decrease in EE, with an average reduction of 0.85 Likert points (*p* = 0.009), compared to psychotherapists who do not conduct online therapy.

## 4 Discussion

The objective of this study was to investigate workplace stress in a sample of psychotherapists: we compared the burnout of those who use online therapy with those who do not and we focused on the association with individual sensitivity to environmental influences. Subsequently, we evaluated the link between use of online therapy, environmental sensitivity and burnout.

### 4.1 Assessment of burnout in psychotherapists

The results showed moderate levels of Emotional Exhaustion among psychotherapists. This outcome aligns with previous research regarding individual experiences of stress accompanied by a decline in emotional and physical resources experienced by psychologists in their work with patients (Kercher et al., 2024; Probst et al., 2020).

The analysis conducted so far has shown low levels of Depersonalization among the survey participants. This finding suggests that most psychotherapists have maintained empathy and connection with their clients.

It was also found that the experience of the pandemic and the current crisis affecting the mental health sector did not impact feelings of professional efficacy and productivity at work: participants exhibited high levels in the dimension of Professional Accomplishment.

### 4.2 Comparison of burnout in traditional and digital psychotherapists

Comparing psychotherapists who practice online therapy and those who do not. It revealed significant results for Emotional Exhaustion and Depersonalization.

Regarding Emotional Exhaustion, it is known that exercising sensitivity, the effort to attune with the patient, and the co-construction of a relationship between partners are necessary to effectively conduct psychotherapeutic treatment. A profound and continuous emotional effort is therefore required of the clinician (Békés et al., 2021; Fernández-Álvarez and Fernández-Álvarez, 2021). The online methodology appears to have had a protective role for the psychotherapist: professionals who did not use it were found to be more emotionally exhausted, increasing the risk of poor outcomes in face-to-face work with the patient (Patel et al., 2018).

Even more advantageous was the use of online therapy for the other dimension of burnout: Depersonalization (the phenomenon of detachment and a cynical attitude of the worker towards their occupation). Participants practicing online consultations demonstrated significantly lower Depersonalization scores compared to those practicing traditional consultations. This result appears to be of some importance: professionals who lose enthusiasm and passion for their occupation with using traditional therapies could expose the treatment to the risk of poor therapeutic outcomes (Singh et al., 2020).

Although it may seem counterintuitive, traditional psychotherapists may feel like an external observers of patients’ mental processes during face-to-face clinical work. “Depersonalized” psychotherapists may experience sensations of coldness, irritation, and negativity in the concrete relationship with patients. This could limit the quality of their performance; for example, they may provide evasive responses to patients’ requests for help, underestimate their problems, and fail to recognize or deny their psychological needs. The therapist’s detachment from the patient, combined with emotional numbness or loss of reactivity, could thus cause discomfort for both partners of the dyad (Yang and Hayes, 2020).

The paradox of depersonalization might, therefore, be conceptualized as a form of coping strategy, a protective mechanism against personally experiencing patients’ lived experiences and excessive identification with them.

In contrast, the digital setting promotes engagement in a deep “disembodied” relationship with the patient. The screen would not constitute a concrete barrier separating therapist and patient; rather, it would function as a metaphorical boundary that regulates distance, stimulates emotional sharing, and facilitates the management of transference-countertransference movements. A “disciplined subjectivity,” balanced between approaching and withdrawing, would favor the therapist’s emotional resonance, mirroring, and their shared experience or participation in the other’s lived experiences, which are essential elements for finding the patients (Zavattini, 2014).

In contrast to various technological tools that can be employed in remote clinical practice, such as text messages, emails, and telephone calls (Zhou et al., 2020), videoconferencing allows the clinician to deeply attend to the patient’s non-verbal cues within a secure environment.

Simpson et al. (2021) discussed the “democratizing effect” in remote treatment. Both therapists and patients share their personal environments, creating a shift in the power balance. In the online setting, the parity between client and therapist would be greater compared to the traditional setting, as both are situated in independent spaces, communicating through their respective technological tools, over which they have individual control (Mitchell, 2020; Norwood et al., 2018). Consequently, patients might feel less threatened by the therapist, their psychological defenses could lower, and patient empowerment would increase, as they would not perceive themselves as submitting to the therapists’ will (e.g., in terms of attending their building, being seated in their room, according to their wishes) (Simpson et al., 2021).

Thus, in virtual reality, conceptualized as a “transitional space” (Jesser et al., 2022), something unique could occur: not only greater openness from the patient but also an empathic and “synchronous” understanding by the therapist (Boldrini et al., 2020). Indeed, it is not physical proximity that is decisive for feeling close and connected, but rather the realization of a “true encounter” (Manguel, 2019).

Research on strategies to combat burnout suggests the need for therapists to disengage from their occupation at the end of work (O’Connor et al., 2018). In this sense, digital mental health ensures greater workplace flexibility for the psychotherapist compared to standard therapy (James et al., 2022). The possibility of working from any location and at any time can mitigate clinical stress, improving the balance between professional and personal life (Olwill et al., 2021).

Moreover, the ease of organizing online meetings with the patient favors continuity in treatment (Yellowlees et al., 2020). The difficult work of building the therapeutic alliance is thus facilitated through continuous online care (Stefana et al., 2024).

These results suggest that the practice of teletherapy may serve as a protective factor in reducing psychotherapists’ burnout, facilitating the development of clinical reasoning and promoting the profound engagement with the patient.

Understanding the interaction between burnout and the practice of online therapy is crucial to support the wellbeing of clinicians and thus ensure quality and effective care for patients.

### 4.3 Burnout and psychotherapists’ sensitivity to environmental influences

In this study, psychotherapists with high levels of Ease of Excitation and Low Sensory Threshold were both strongly and negatively affected by potentially stressful experiences, with an associated increase in Emotional Exhaustion. While less sensitive individuals tend to be more robust and resilient when facing adversity, highly sensitive individuals are more reactive to adverse experiences and tend to adopt less adaptive coping strategies (Belsky and Pluess, 2009).

However, the findings also proposed a more nuanced picture, suggesting that certain aspects of environmental sensitivity may be beneficial for personal accomplishment. Psychotherapists with heightened Aesthetic Sensitivity may experience a greater sense of mastery over situations, self-determination and self-efficacy in their work, possibly due with their ability to process environmental influences in a deep and detailed manner (Redfearn et al., 2020; Sun et al., 2023).

These results highlight the complex relationship between individual personality and occupational wellbeing. Differences in the degree of psychotherapist burnout could be related to the complex and distinctive set of characteristics and psychological processes that define the personality (Van Hoy and Rzeszutek, 2022). High environmental sensitivity has emerged as a significant factor associated with psychotherapist burnout, functioning as both a potential risk factor and a possible protective factor.

### 4.4 Link between online therapy use, environmental sensitivity and burnout

The moderation models revealed the specific utility of the online methodology, particularly for psychotherapists with higher environmental sensitivity. For these individuals, the use of remote consultations was associated with lower emotional exhaustion compared to the other respondents who do not practice online consultations.

In line with vantage sensitivity framework, more sensitive psychotherapists would be more reactive to the positive effect of teletherapy compared to less sensitive psychotherapists (De Villiers et al., 2018).

Significant results were found in the dimensions Ease of Excitation and Low Sensory Threshold of Environmental Sensitivity.

Regarding Ease of Excitation, online therapy use showed a stronger association with lower Emotional Exhaustion of psychotherapists who are more reactive to external stimuli compared to less excitable psychotherapists who do not use online therapy.

Highly reactive psychotherapists would feel overwhelmed in highly stimulating work environments. Public health services, particularly psychiatric hospitals and mental health centers, exemplify such environments, exposing professionals to intense demands (Giusti et al., 2020; Luo et al., 2020). Psychotherapists working in these settings are required to respond quickly and appropriately to patient needs, often managing delicate situations in hectic conditions (Mittal et al., 2023). In such chaotic environments, clinicians prone to excitation would experience discomfort, stress and fatigue. The online setting reduces exposure to excessive stimulation (Stoll et al., 2020).

Regarding Low Sensory Threshold, psychotherapists with heightened sensibility to stimuli seemed to benefit from online practice in terms of reducing Emotional Exhaustion compared to those who do not conduct online therapy. Their heightened sensitivity to low-intensity stimuli can make them particularly susceptible to environmental changes.

In this regard as well, teleconsultations with patients would benefit clinicians working in public health settings, which are subject to frequent and often unpredictable environmental variations. Teletherapy thus constitutes a controlled alternative where sensory input can be minimized and the expression of practitioners’ clinical skills can be facilitated, helping them to buffer the stressors of traditional face-to-face settings (Batastini et al., 2021; Rutkowska et al., 2023).

### 4.5 Limitations

This study presents several limitations.

The complexity of the survey and the time required to complete the questionnaires reduced the number of participants. Although the survey included various professionals working in the field of mental health, the majority of participants were psychologists. Furthermore, the study was conducted during a period when most respondents were directly involved with patients, which may have limited survey participation. These limitations make it difficult to generalize the results.

The cross-sectional nature of the study and the lack of longitudinal follow-up do not allow for inferences about causal relationships between variables and the long-term consequences of the psychological outcomes observed. These limitations preclude solid conclusions.

Finally, qualitative studies could explore in depth the individual experiences of psychotherapists adopting digital methodologies to thoroughly understand the effects of different digital spaces on the psychotherapeutic process and expand research on the possibilities and limitations of virtual encounters for psychological maturation. Further research is needed to carefully investigate the “complex mixture of proximity and distance, presence and absence, reality and fantasy” (Roesler, 2017) that characterizes exchanges in the digital setting, creating new forms of relationships that differ significantly from traditional face-to-face interactions.

## 5 Conclusion

This study provides a snapshot of the benefits of online therapy practice in reducing workplace stress among psychotherapists, with significant implications for the future healthcare policies and the clinical method in psychotherapy.

Teletherapy is particularly useful for enhancing the wellbeing of psychotherapists with high environmental sensitivity, which can be measured with a brief questionnaire. In this way, strategic *ad hoc* choices, based on individual differences, could be implemented in different clinical settings. One solution involves the targeted use of online therapy by more sensitive clinicians in particularly stimulating contexts, including psychiatric hospitals and mental health services. The virtual dimension of the online setting, isolated and detached from the concreteness of the physical environment, would reduce exposure to intense stimuli and allow for greater control over the therapeutic setting.

Digital tools can represent interesting, useful, and effective resources to integrate into the clinical method in psychotherapy. Online consultation does not replace conventional treatment but serves as a valuable complement to psychological practice. Therefore, mental health organizations should invest in digital health by providing resources to support the tele-psychotherapy. Most clinicians have little education in the online practice (Doan et al., 2021; Perle et al., 2024). It would be useful to promote training programs, guidelines and best practices to effectively use digital platforms and transition patient care to an online format. Technological development in the field of mental health predicts future trends that include “smart” mobile devices, cloud computing, virtual worlds and realities, electronic games, in addition to conventional psychotherapy tools. Greater knowledge of these tools and their main differences is necessary to adapt them to the specific purposes of the clinician.

It is beneficial to support a more efficient “connected” healthcare system by strengthening care and replicating successful practices, also in preparation for possible future emergencies.

In conclusion, the development of socially advantageous psychotherapy strategies continues to represent a global challenge in this post-COVID period. This study leverages advances in digital health to propose personalized healthcare solutions aimed at promoting the wellbeing of psychotherapists, providing adequate responses to the bio-psycho-social treatment needs of patients and enhancing the quality of care in different clinical settings.

## Data Availability

The raw data supporting the conclusions of this article will be made available upon request by the authors, without undue reservation.
